# Supplementary material for: Environmental Factors Associated with Physical Activity in Rural U.S. Counties
Source: Int J Environ Res Public Health. 2021 Jul 20;18(14):7688. doi: 10.3390/ijerph18147688 (PMC8307667; doi:10.3390/ijerph18147688)
Supplement: Supplementary file 1 [file ijerph-18-07688-s001.zip › ijerph-1302142-supplementary.pdf]

Supplementary Table S1. Bivariate Correlations for Male and Female Physical Activity by Natural Environment Indicators

		All Groups (n= 2679)							Urban (n= 565, 0-25% rural)							Somewhat Urban (n= 573, 25-48% rural)						
Indicator		1	2	3	4	5	6	7	1	2	3	4	5	6	7	1	2	3	4	5	6	7
1	Male PA	-							-							-						
2	Female PA	.87	-						.86	-						.82	-					
3	Air Temperature	-.48	-.54	-					-.35	-.41	-					-.53	-.59	-				
4	Heat Index*	-.48	-.52	.69	-				-.47	-.53	.62	-				-.55	-.59	.70	-			
5	Precipitation	-.21	-.26	-.13	.09	-			.03	-.04	-.31	.08	-			-.06	-.20	-.13	.13	-		
6	Sun	-.23	-.29	.85	.34	-.37	-		-.14	-.17	.84	.22	-.53	-		-.25	-.28	.81	.27	-.41	-	
7	Water*	.21	.19	-.11	-.13	.04	.04	-	.10	.07	-.02	-.02	.18	.12	-	.15	.15	-.07	-.10	.10	.02	-
		Somewhat Rural (n= 577, 48-67% rural)							Rural (n= 577, 67-99% rural)							Exclusively Rural (n= 387, 100% rural)						
Parameter		1	2	3	4	5	6	7	1	2	3	4	5	6	7	1	2	3	4	5	6	7
1	Male PA	-							-							-						
2	Female PA	.85	-						.87	-						.92	-					
3	Air Temperature	-.57	-.62	-					-.56	-.61	-					-.53	-.60	-				
4	Heat Index*	-.50	-.53	.74	-				-.42	-.46	.76	-				-.34	-.37	.70	-			
5	Precipitation	-.17	-.23	-.15	-.01	-			-.30	-.30	-.09	-.03	-			-.51	-.51	.17	.14	-		
6	Sun	-.38	-.43	.88	.44	-.36	-		-.39	-.45	.91	.55	-.24	-		-.33	-.42	.87	.39	-.04	-	
7	Water*	.20	.17	-.21	-.17	.04	-.12	-	.30	.30	-.24	-.19	-.15	-.07	-	.12	.11	-.10	-.15	-.03	.06	-

Notes: *= indicator required a log transform using the following equation: $\mathrm{LOG1PX}(x) = \log(1 + x)$

Supplementary Table S2. Bivariate Correlations for Male and Female Physical Activity by Social Environment Indicators

	All Groups (n= 2577)	Urban (n= 571, 0-25% rural)	Somewhat Urban (n= 561, 25-48% rural)

	Indicator	1	2	3	4	5	6	1	2	3	4	5	6	1	2	3	4	5	6
1	Male PA	-						-						-					
2	Female PA	.87	-					.86	-					.82	-				
3	Alcohol Vehicle Deaths	.08	.08	-				.04	.06	-				.10	.09	-			
4	Violent Crime*	-.10	-.19	.04	-			-.27	-.34	.01	-			-.32	-.41	.01	-		
5	Single Parent Households	-.36	-.47	.03	.50	-		-.45	-.54	.01	.69	-		-.42	-.52	.02	.57	-	
6	Vacant Housing*	-.15	-.15	.07	.02	.25	-	-.17	-.22	-.02	.34	.44	-	.06	.05	.09	.24	.26	-
		**Somewhat Rural (n= 554, 48-67% rural)**						**Rural (n= 554, 67-99% rural)**						**Exclusively Rural (n= 337, 100% rural)**					
	Parameter	1	2	3	4	5	6	1	2	3	4	5	6	1	2	3	4	5	6
1	Male PA	-						-						-					
2	Female PA	.85	-					.87	-					.93	-				
3	Alcohol Vehicle Deaths	.05	.05	-				.17	.16	-				.05	.05	-			
4	Violent Crime*	-.30	-.41	.08	-			-.22	-.29	.01	-			-.17	-.25	.11	-		
5	Single Parent Households	-.46	-.59	.07	.60	-		-.26	-.38	.01	.46	-		-.40	-.51	.05	.33	-	
6	Vacant Housing*	-.05	-.10	.13	.31	.40	-	.22	.18	.10	.17	.25	-	.08	.01	.06	.28	.20	-

Notes: *= indicator required a log transform using the following equation: LOG1PX(x) = l o g (1 + x)

Supplementary Table S3. Bivariate Correlations for Male and Female Physical Activity by Recreation Environment Indicators

		**All Groups (n= 2686)**						**Urban (n= 576, 0-25% rural)**						**Somewhat Urban (n= 576, 25-48% rural)**					
	Indicator	1	2	3	4	5	6	1	2	3	4	5	6	1	2	3	4	5	6
1	Male PA	-						-						-					
2	Female PA	0.87	-					0.86	-					0.82	-				
3	Access to Exercise Opportunities	0.45	0.46	-				0.30	0.32	-				0.31	0.35	-			

Indicator	1	2	3	4	5	6	1	2	3	4	5	6	1	2	3	4	5	6
4 Live within 150 Miles of Highway	0.19	0.15	0.36	-			-0.10	-0.13	0.19	-			0.05	0.04	0.12	-		
5 Live within Half-Mile of Park	0.43	0.44	0.51	0.33	-		0.22	0.27	0.55	0.17	-		0.25	0.34	0.35	0.03	-	
6 Elementary Half-Mile	0.23	0.27	0.20	0.21	0.54	-	-0.13	-0.04	0.20	0.27	0.50	-	-0.02	0.11	0.13	0.07	0.47	-

	**Somewhat Rural (n= 576, 48-67% rural)**						**Rural (n= 577, 67-99% rural)**						**Exclusively Rural (n= 381, 100% rural)**					
Indicator	1	2	3	4	5	6	1	2	3	4	5	6	1	2	3	4	5	6
1 Male PA	-						-						-					
2 Female PA	0.85	-					0.87	-					0.93	-				
3 Access to Exercise Opportunities	0.43	0.47	-				0.36	0.37	-				0.25	0.29	-			
4 Live within 150 Miles of Highway	0.04	0.06	0.14	-			0.08	0.06	0.08	-			0.00	-0.04	0.06	-		
5 Live within Half-Mile of Park*	0.25	0.32	0.25	0.05	-		0.36	0.38	0.24	0.00	-		0.39	0.41	0.15	0.05	-	
6 Elementary Half-Mile	0.08	0.18	0.04	-0.03	0.43	-	0.26	0.29	0.04	-0.02	0.48	-	0.36	0.38	-0.06	0.10	0.43	-

Notes: *= indicator required a log transform using the following equation: $LOG1PX(x) = \log(1 + x)$

Supplementary Table S4. Bivariate Correlations for Male and Female Physical Activity by Transportation Environment Indicators

	**All Groups (n= 2697)**							**Urban (n= 577, 0-25% rural)**							**Somewhat Urban (n= 577, 25-48% rural)**						
Indicator	1	2	3	4	5	6	7	1	2	3	4	5	6	7	1	2	3	4	5	6	7
1 Male PA	-							-							-						
2 Female PA	0.88	-						0.86	-						0.82	-					
3 Bike to Work*	0.45	0.47	-					0.44	0.50	-					0.45	0.51	-				
4 Walk to Work*	0.31	0.36	0.44	-				0.14	0.18	0.54	-				0.34	0.41	0.57	-			
5 Works in County	0.19	0.18	0.37	0.36	-			-0.05	-0.07	0.26	0.24	-			0.13	0.16	0.37	0.43	-		

6 Works in Place*	0.18	0.16	0.35	0.36	0.65	-		-0.21	-0.20	0.28	0.36	0.55	-		-0.01	0.08	0.30	0.39	0.67	-	
7 Long Commute	-0.15	-0.16	-0.32	-0.34	-0.74	-0.66	-	0.20	0.18	-0.16	-0.28	-0.63	-0.59	-	-0.01	-0.09	-0.34	-0.42	-0.76	-0.68	-

	**Somewhat Rural (n= 577, 48-67% rural)**							**Rural (n= 577, 67-99% rural)**							**Exclusively Rural (n= 389, 100% rural)**						
Indicator	1	2	3	4	5	6	7	1	2	3	4	5	6	7	1	2	3	4	5	6	7
1 Male PA	-							-							-						
2 Female PA	0.85	-						0.87	-						0.93	-					
3 Bike to Work*	0.33	0.37	-					0.28	0.29	-					0.37	0.37	-				
4 Walk to Work*	0.28	0.35	0.35	-				0.44	0.46	0.35	-				0.36	0.41	0.34	-			
5 Works in County	0.08	0.09	0.26	0.34	-			0.15	0.10	0.26	0.42	-			0.16	0.22	0.26	0.43	-		
6 Works in Place*	-0.01	0.03	0.22	0.37	0.58	-		0.12	0.06	0.19	0.37	0.57	-		0.19	0.19	0.12	0.44	0.53	-	
7 Long Commute	-0.03	-0.10	-0.26	-0.31	-0.75	-0.57	-	-0.23	-0.20	-0.24	-0.34	-0.71	-0.51	-	-0.21	-0.25	-0.29	-0.37	-0.73	-0.60	-

Notes: *= indicator required a log transform using the following equation: LOG1PX(x) = l o g (1 + x)
